# Vis Medicatrix Naturæ

**Published:** 1889-08

**Authors:** J. W. Harrison

**Affiliations:** Dripping Springs, Texas


DANIEL'S

### TEXAS MEDICAL JOURNAL.

A Representative Organ of the Medical Profession, and an Exponent of Rational Medicine; devoted to the Organization, Advancement and Elevation of the Profession in Texas.

PUBLISHED MONTHLY.—SUBSCRIPTION $2.00 A YEAR.

Vol. V. AUSTIN, AUGUST, 1889. No. 2.

### ORIGINAL ARTICLES.

CONTRIBUTED EXCLUSIVELY TO THIS JOURNAL.

*The Articles in this Department are accepted and published with the understanding that we are not responsible for, nor do we indorse the views and opinions of the writers by so doing.*

### VIS MEDICATRIX NATURÆ.

BY J. W. HARRISON, M. D., DRIPPING SPRINGS, TEXAS.

Read at Austin District Medical Association, and ordered published in the official organ of the Society.

WE NOW BEGIN another year of search after that more complete knowledge of diseases, their treatment and their cure, which every true physician aspires to; and while we may never attain to perfection in our science, we can, by study and research, acquire a more perfect understanding of those hidden mysteries in diseased nature, which it would be both our duty and delight to make plain, and many of them will be, if we but continue these meetings and each member contribute something of what he has learned from his observations and his ministrations to the sick. And it is to be hoped that every member of our Society will be free to express his thoughts on every subject which may be presented for consideration; for while some may not have attained to that degree of eminence in the science of medicine which others have, still, from their vast store of practical knowledge, gathered by the bedside of the sick and the dying, there is not one who may not drop one thought, make one suggestion that will be of untold benefit to his confreres in the healing art. And who of us has not experienced the time when we would have gladly availed ourselves of the advice and counsel of the most humble? So to-day I offer my mite towards enhancing the object aimed at by these meetings. I have chosen for my theme "The Vis Medicatrix Naturæ," a subject fraught with much interest to us and to mankind generally; and while I may not offer anything new to you, I shall have served a good purpose if I but arouse in your minds a greater desire and determination to know more about that power in the system which we call the *Vis Medicatrix Naturæ.*

That this curative power or property does exist in the living animal body, none will deny. And while the idea may have been carried needlessly far, and rendered, perhaps, too material, it is still a subject that concerns us deeply, being, as one of the ancient writers terms it, "The Archeus, or grand regulator of the human body." In your weary professional rounds, there is never a day but brings additional proof that there is in the constitution a power to repair injuries and to restore lost parts; and that actions, which we call diseases, are instituted to get rid of the causes which produce them. Nor is there any necessary extravagance in the supposition that all may be. The law may perhaps be said to be analogous, or conformable to, the law of which natural philosophers have gained a knowledge, by induction, from various phenomena—that all *action* is followed by *reaction* equal to, but different from, the primary action. We know that if a bone be broken, if a muscle or tendon or even a nerve be divided, the divided ends will unite. If a portion of substance, as of skin and cellular membrane and muscle, is lost or cut out, the deficiency is repaired and the cavity filled up by granulations from the bottom, and at last covered in by new skin. If a part be removed, and is without delay repaired, (for instance, a tooth, the tip of the ear, or finger, or a part of the nose,) it becomes firmly united in its original situation. So anxious (if you will allow the expression) is nature to preserve the life she has given, that wounds of the most important organs will cicatrize, wounds of the lungs, of the heart, of the bladder, of the intestines, of the uterus, even of the brain.

As among the salutary processes of nature, some writers have classed shivering, convulsions, sighing, gout, fever wasting diseases, dispepsia, vertigo, and loss of appetite. The propriety of so considering some of these, may be doubted; but illustrations of the salutary effects of the majority of them will so readily present themselves to those familiar with pathological phenomena as to require no further observation. Viewing the principle in its widest extent, it may be said to be first exemplified in the convulsions of infancy, and it is exhibited for the last time, and so often ineffectually, in the convulsions so frequently preceding death. There is a consolation, not unattended with a hope of the final extension of medicine far beyond its present powers, to be derived from observing that even diseases of a most fatal character evince a conformity (as it were) to the same great curative and preventive law. The whole series of the changes in the tubercle are but so many efforts to dislodge and eliminate it from the lungs; the process is attended with irritation, and by long continuance, or rather by constant repetition, generally ends in death. A similar process prevails in cancer, and is sometimes, although rarely, more happily effected. Calculi in the bladder become enveloped in pouches of the lining membrane and irritate the patient no more, and sometimes escape by ulceration into the rectum or vagina, or through the abdominal integuments.

There can be no difficulty in admitting these indications of the "Vis Medicatrix Naturæ," without referring them to anything more than an internal sense, which the preservation of existence did not require to be of the same nature as what are called the *external* senses. It would hardly be necessary to the more material character given to the power, if the physicians who were most disposed to invest it with personal properties and dignity had not, as a natural consequence, become too scrupulous about opposing it, even to the extent of curing all diseases by what the French have called "Expectation." The proper advantage of admitting such a power is, that it prevents indiscriminate and officious meddling, that "Medicina purturbatrix," which incessantly contradicts even the best efforts towards a natural cure. In the modern practice of medicine and surgery, the "vis medicatrix" is seldom entirely disregarded, but its suggestions are not followed without discretion, and never a case presents itself but what the question forces itself upon us, "How far should we trust that unquestionable power?" For while we must all acknowledge the intention of its working, we often see it work in a very dangerous manner, and too much by a general rule; sometimes too abruptly, sometimes after too lingering a manner. The prime object of art is to adapt the rule to particular instances; and the greatest wisdom of the practitioner is shown in deciding when to wait upon nature, when to rouse her to action, when to moderate those exertions, and when to put an absolute stop to them.

When a bone is broken (the body being in full health), the vis medicatrix is seen in full activity—the whole restorative force is called out. The vascularity, the heat, the vitality of the surrounding parts is increased, and inflammation ensues. But it is often necessary to moderate this zeal and haste of nature,—to lessen the superfluous excitement, leaving only that degree of it without which the repair cannot proceed. The savages trust to the natural power of healing so entirely, that it is said the most frightful wounds will heal without surgical aid. On the other hand, a patient of infirm constitution or disordered health, becomes subject to some local congestion, or some accidental or trifling injury brings a part of the integuments into a state of chronic or inactive disease, and the "Vis Medicatrix Naturæ" is not exerted until the obstacle to its exertion, the disordered state

(perhaps of the digestive organs) is removed by alterative medicines. But that done, and a wound or an abrasion which had remained stationery for weeks, instantly puts on a new character. Secretory and absorbant processes are at once commenced, and the cure is soon completed. We see, too, in the case of a patient alarmingly lowered by an attack of fever, the nervous energy sunk, the power of the heart diminished, the digestive function almost annihilated, the respiration laborious and feeble, nature, unassisted, will try to make this patient rally; but if successful, it can only be done in the course of many weeks, perhaps months, and the body and mind will long continue to suffer, or perhaps never wholly recover. But we know that by the timely use of stimulants and tonics we can arouse the powers of digestion, and reanimate the whole frame in less than half the time, and with less than half the danger. Whilst, however, we seriously consider the design of nature in any malady, whether she is working and how she is working, and how the machinery will endure the process she has set up, we should still never trust entirely to the "Vis Medicatrix Naturæ," except in cases of the most trifling injuries, where a process of very small extent is all that is required for the cure.

The natural powers of healing and recovery are in many cases strongly influenced by peculiar mental states. The same emotions and passions which redden and blanch the cheek, which affect the cutaneous or renal functions, or those of the stomach and bowels, can also promote or retard the activity of the "Vis Medicatrix Naturæ," and for that reason the question again presents itself, "How far should physicians trust to that power of nature?"

Confidence of recovery often conduces to recovery and extreme despondency as often to fatal results. This is very conspicuous in some affections, particularly in fevers, and has been asserted by surgeons to prevail in malignant disorders, especially in cancer, and therefore, some practitioners are inclined to place implicit reliance on the prognostics, in which their patients are disposed to indulge. And while I believe that is rather to extremes, the influence of mind over disease should never be overlooked by the physician, nor should he forget to teach the fact, that this influence of the mind, like all the mental actions, may by firm endeavor be, to a great extent, subjected to the will.

Of this influence we have a very striking example in the many and surprising recoveries resulting from the so-called "Christian Science" and "Mind Cure." And is not the relation of the "mind cure" movement to ordinary medical practice very important, in that it emphasizes what the most philosophical physicians of *all* schools have always deemed of the first importance: that medicine is but occasionally necessary? Of that fact we have abundant proof in the large number of cases, grave cases, which recover without medical aid, simply trusting to the "vis medicatrix naturæ." Doctors, are we not too vain of our knowledge of "materia medica;" and does not that vanity often lead us to prescribing medicines, when they might often be dispensed with entirely, much to the advantage of the patient? Who does not know that the day of massive pills and bulky powders has long since past and gone? And what physician of any intelligence or progress in the science of medicine will not confess that the tendency of the time is toward less and simpler medicine and a greater reliance upon the "vis medicatrix naturæ?" which for years has been acknowledged by the leaders of the medical profession, as the final element in every cure. Sir John Forbes, one of the most prominent regular practitioners of England, in his famous article on homeopathy, says: "First, that in a large portion of the cases treated by allopathic physicians, the disease is cured by nature and not by them; second, that in a lesser, but still not a small proportion, the disease is cured by nature in spite of them; third, that consequently, in a considerable portion of diseases, it would fare as well, or better, with patients, if all remedies, at least, active remedies, especially drugs, were abandoned."

Even as long ago as Sydenham's time, that great scholar and teacher said: "I often think that more could be left to nature than we are in the habit of leaving to her; to imagine that she always wants the help of art, is an error, and an unlearned error too."

Sir John Marshall, of the London University school, in his opening address to the session of 1865, said: "The vis medicatrix is the agent to employ in the healing of an ulcer or the union of a broken bone; and it is equally true that the physician or surgeon never cured a disease; he only assists the natural process of cure, performed by the intrinsic, conservative energy of the frame, and this is but the extension of force imparted at the origination of the individual being."

So we see, that in the days long since gone by, when the medical science was obscured by the darkness of the middle ages, even down to the present day of wonderful enlightenment and progress, physicians advocated a greater reliance upon the "vis medicatrix naturæ." But, unfortunately, they did not have, nor have they yet, a proper faith in nature's power to heal, a faith that would lead them to let her severely alone, except in those instances in which she was either too slow, or too weak to accomplish the end aimed at. With the knowledge which we have of her ability to heal, does it not behoove us, her coadjutors in the divine art, to advocate a greater reliance upon "vis medicatrix naturæ?" I believe the day is not far distant, when most *sensible* persons will prefer a physician who understands both mind and body, who can be a "father confessor" to the sick man, relieving him of the responsibility of treating himself, quieting his mind, and strengthening him by hope, and stimulating him by his personal presence; one, who, understanding the mineral, plant and animal substances included in the materia medica, can assist nature, interfering only when absolutely necessary and certainly safe; too learned and honest when not knowing what to do, ever to do he knows not what.

They will also prefer a physician who can relieve their pains, when incurable; smooth their pathway to the inevitable end; or when he has the happiness to see them convalescent, will be able to give him such hygienic hints as may prevent the recurrence of the malady, or save them from something worse. So, let us in these meetings, while striving to learn more of diseases and medicines and appliances which conduce most to their alleviation and cure, give more thought to the "vis medicatrix naturæ?" Can it be trusted? When, and how far? And when we have solved that problem, let us pursue the even tenor of our way, trusting that the verdict of mankind (excepting minds prone to vagaries on the borderland of insanity) will be that pronounced more than two thousand years ago by Ecclesiastic:

"The Lord hath created medicines out of the earth, and he that is wise will not abhor them. My son, in thy sickness be not negligent, but pray unto the Lord and he will make thee whole. Leave off from sin and order thy hands aright; and cleanse thy heart from all wickedness. Then give place to the physician, for the Lord hath created him. Let him not go from thee, for thou hast need of him. There is a time, when in their hands there is good success. For they shall also pray unto the Lord that he would prosper that which they give for ease and to prolong life."

